# Comparison of immunosuppressive therapies for IgA nephropathy after tonsillectomy: three-course versus one-course steroid pulse combined with mizoribine

**DOI:** 10.1007/s11255-015-1118-6

**Published:** 2015-10-03

**Authors:** Tomohiro Kaneko, Momoko Arai, Mariko Ikeda, Megumi Morita, Yoko Watanabe, Akio Hirama, Akira Shimizu, Shuichi Tsuruoka

**Affiliations:** Division of Nephrology, Department of Internal Medicine, Nippon Medical School, 1-1-5 Sendagi, Bunkyo-ku, Tokyo, 113-8603 Japan; Department of Analytic Human Pathology, Nippon Medical School, Tokyo, Japan

**Keywords:** IgA nephropathy, Tonsillectomy, Steroid pulse, Mizoribine

## Abstract

**Purpose:**

It has been reported that steroid pulse therapy for IgA nephropathy improves renal prognosis. However, because of the side effects, steroid dose must be restricted to some cases. Treatment effects of steroid on cases already presenting with reduced renal function are unknown. In this study, we performed tonsillectomy in patients with IgA nephropathy and conducted a comparative study about subsequent immunosuppressive therapy.

**Methods:**

Subjects were patients younger than 70 years of age diagnosed with IgA nephropathy by renal biopsy. Treatment protocols were a single-course steroid pulse combined with mizoribine during a period from August 2006 to June 2010 (Group A; *n* = 34) and a three-course steroid pulse during a period from July 2010 to March 2013 (Group B; *n* = 32). Primary end points were excretory amounts of proteinuria, disappearance of proteinuria and hematuria, and exacerbation of renal function.

**Results:**

In both the groups, proteinuria decreased significantly 12 months after treatment, and no significant difference in alleviation effects on proteinuria was found between groups. eGFR increased significantly 12 months after treatment in Group A, whereas it tended to decrease in Group B. As for the preservation effect on eGFR, Group A showed significantly higher preservation of eGFR. Similar results were shown in the patients whose eGFR at the start of the treatment was less than 60 mL/min/1.73 m^2^.

**Conclusions:**

Single-course steroid pulse therapy combined with mizoribine was considered to have a protective effect on the renal function in IgA nephropathy, especially accompanying renal dysfunction.

## Introduction

IgA nephropathy was initially reported by Berger and Hinglais in 1968 [1]. Since then, a great deal of research regarding its etiology, pathologic conditions, and establishment of treatment methods has been presented. Its clinical presentation is various, including natural remission cases, gradual progressions to renal failure in the terminal stage, and cases of rapid exacerbation of renal function. This varied pathologic condition has prevented establishment of specific treatment methods.

There have been many reports which state that steroid treatments are effective for inhibiting the progress of IgA nephropathy [2, 3]. However, in a randomized controlled trial (RCT) in which treatment using an oral steroid of 20 mg/day for 2 years in patients with IgA nephropathy whose serum creatinine level was less than 1.5 mg/dL, although reduced proteinuria was found progress of renal dysfunction was not inhibited [4]. Pozzi et al. [5] conducted a multicenter study in subjects with IgA nephropathy having proteinuria of 1.0–3.5 g/day and serum creatinine levels of 1.5 mg/dL or less and reported that steroid therapy, including three-course pulse therapy, significantly inhibited the progress of renal dysfunction up to 10 years after treatment. However, they also showed some cases where proteinuria recurred after treatment and the need to give the same scheme occurred.

Combined treatment of steroid pulse therapy with bilateral tonsillectomy has been performed mainly in Japan. There have been numerous reports demonstrating a high remission rate obtained by this treatment in early-stage cases [6–8]. Recently, a multicenter prospective study in IgA nephropathy cases with conditions identical to those in Pozzi et al. was conducted. Compared with three-course steroid pulse treatment only, steroid pulse therapy combined with tonsillectomy showed no significant difference in remission of the urinary findings, but 12 months following treatment resulted in significant reduction in proteinuria [9].

In cases where glomerular filtration rate (GFR) has already decreased, increases in sclerotic glomerulus as well as progress of interstitial fibrosis are pointed out with the high dose of steroid. Thus, in treating IgA nephropathy in CKD stage 3 or greater, renin–angiotensin system (RAS) inhibitors have been primarily used. However, effects of RAS treatment alone are insufficient. On the other hand, in the patients with progressive IgA nephropathy with serum creatinines of 1.5 mg/dL or higher, there has been a report stating that RCT by immunosuppressive therapy with oral steroids and cyclophosphamide and azathioprine decreased proteinuria and hematuria and inhibited the progress of renal dysfunction [10].

We previously reported that in IgA nephropathy with dominant chronic lesions and additional acute active lesions, after tonsillectomy, single-course steroid pulse therapy combined with mizoribine (MZR) resulted in improvement of the renal function [11, 12]. However, differences in the treatment effects of different types of conventional steroid pulse therapy combined with tonsillectomy have not been clarified.

To answer this, 34 cases of single-course steroid pulse therapy + MZR and 32 cases of three-course steroid pulse therapy after tonsillectomy were compared for alleviation in hematuria and proteinuria and inhibitory effects in progress of renal dysfunction.

## Materials and methods

### Patients

Criteria for steroid pulse therapy combined with tonsillectomy at our hospital are as follows: ① patients who are diagnosed IgA nephropathy by renal biopsy, ② patients who present persistent microscopic hematuria and proteinuria, ③ patients whose estimated value of GFR (eGFR) is 20 mL/min/1.73 m^2^ or higher, ④ patients younger than 70, ⑤ patients where general anesthesia is possible, and ⑥ ability and willingness to provide informed consent. Exclusion criteria are: patients with a history of anaphylaxis drugs, who have renal lesions resultant from systemic diseases such as SLE, who are pregnant or may be pregnant, who have difficulty controlling blood pressure, or who are contraindicated for tonsillectomy as assessed by otolaryngologist.

As a rule, following tonsillectomy, all the patients whose treatment began during the period of August 2006–June 2010 received a protocol of single-course steroid pulse therapy combined with MZR (Group A), and all the patients whose treatment started during the period of July 2010–March 2013 received a protocol of three-course steroid pulse (Group B). For this analysis, the subjects were selected whose proteinuria at the start of the treatment was 0.5 g/g Cr or higher and could be followed up for 12 months. This study was prospective controlled study, but not randomized one. The treatment protocol was fixed by the period, and informed consent was described all of factors related this study completely. After treatment of Group A was finished, we got the approval of the ethical committee for this controlled study. The treatment of Group B was provided under a condition same as Group A. The ethical number of our institutional research committee was 23-04-162.

### Therapeutic Intervention

Bilateral tonsillectomy was performed by an otolaryngologist, and 1 week later, methylprednisolone (0.5 g/day) was administered by drip infusion for 3 days. Simultaneously, administration of antiplatelet drugs, antiulcerative drugs, sulfamethoxazole–trimethoprim (ST) combination drugs, and bisphosphonate was started. Before this step, procedures are identical in all the patients.

#### Group A

After methylprednisolone pulse, oral administration of prednisolone (PSL) 30 mg/day was started. Four weeks later, the dose was changed to 30 mg every second day. At the same time, MZR 100–150 mg/day once a day was started. PSL was reduced by 5 mg every 4 weeks and finally discontinued. ST combination drugs and antiulcerative drugs were discontinued at the time point when the dose of PSL became 20 mg every second day. Together with the discontinuation of PSL, bisphosphonate was also discontinued. MZR and an antiplatelet drug were administered continuously for 12 months. Although ordinary oral administration of MZR is three times a day with a 50 mg dose, in this study, as a rule, we administered it once a day with a dose of 150 mg. This was due to the concentration of 1 μg/mL being effective for inhibition of the proliferation of lymphocytes of MZR, and a single administration of 150 mg is appropriate for obtaining a 1 μg/mL blood peak level in humans [13]. The excretion of MZR is a urinary type with 81 % of the unchanged rate in urine, and it is thus required to adjust the dose at the time of renal dysfunction. In cases where the eGFR becomes less than 30 mL/min/1.73 m^2^, peak level may exceed 3 μg/mL to induce hyperuricemia or myelosuppression, which is problematic. Thus, we adjusted the MZR level by measuring blood concentration so as the peak level did not exceed 3 μg/mL.

#### Group B

After the methylprednisolone pulse, administration of PSL 30 mg every second day was started. Two and four months later, the same doses of methylprednisolone pulse were added. Although the protocol of this steroid pulse therapy is nearly the same as a trial conducted by Pozzi et al. [5], the drip infusion dose of methylprednisolone differed. In this study, it was 0.5 g/day. Starting 6 months after tonsillectomy, the dose of PSL was deceased gradually by 5 mg every 4 weeks and finally discontinued. ST combination drugs and antiulcerative drugs were discontinued at the time point when the dose of PSL became 20 mg every second day. Together with the discontinuation of PSL, bisphosphonate was also discontinued. Antiplatelet drugs were administered continuously for 12 months.

To assess whether antihypertensive drugs have an inhibitory effect on RAS, in cases where the drug was already taken when treatment started, the drug was taken continuously and new oral drugs were not allowable for 12 months. If blood pressure rose, other antihypertensives such as Ca antagonist drugs were administered.

### Evaluation of treatment effects (efficacy assessment)

For proteinuria, quantitative determination was conducted as needed by urinary creatinine. A level of less than 0.2 g/g Cr was regarded as remission. For hematuria, a erythrocyte count of less than five by high-power field microscopic examination of urine was regarded as remission. Hematuria severity was scored with urinary dipstick tests. For evaluation of renal function, the glomerular filtration estimation formula for Japanese made by Japan Kidney Association [eGFR = 194 × (sCr)^−1.094^ × (Age)^−0.287^ × (0.739 if female)] was used for the estimation. During the follow-up observation period, blood pressure, general blood examinations, biochemical examinations, and urinalysis, marker examinations of infections were conducted to confirm the presence or absence of appearance of harmful events.

### Statistics

For analysis, normality was tested, and a Wilcoxon rank-sum test was conducted. The uniformity in background variables was analyzed by Chi-squared test, and numerical parameters were analyzed by repeated measures ANOVA for reference. A significance level was determined *p* < 0.05 (bilateral). Microsoft Windows SPSS Ver. 11.0 was used for statistical software. The data were expressed by mean values ± SD.

## Results

Table 1 shows the baseline clinical characteristics of patients at initiation of treatment. Group B has slightly more males, although this was not statistically significant. No significant difference was found between groups for factors that might influence treatment effects, such as number of years from onset to the start of the treatment, proteinuria, or renal function. Four histologic grades, I, II, III, and IV, were established, corresponding to <25, 25–49, 50–74, and ≥75 % of glomeruli exhibiting cellular/fibrocellular crescents, global sclerosis, segmental sclerosis, or fibrous crescents. It was reported this histologic classification could identify the magnitude of the risk of progression to ESRD and was useful for predicting long-term renal outcome in IgA nephropathy [14]. Histologic grade did not have a difference between groups. The rates of taking RAS inhibitors were 50 % in Group A and 44 % in Group B, which were not significant. About the patients with proteinuria more than 1 g/g Cr, the rates of taking RAS inhibitors were 44 % in Group A (*n* = 23) and 47 % in Group B (*n* = 19). All patients with eGFR < 60 mL/min/1.73 m^2^ were taking RAS inhibitors, Group A (*n* = 12), Group B (*n* = 9).

**Table 1:** Baseline clinical characteristics of patients

	Group A One course of steroid pulse + MZR	Group B Three courses of steroid pulse	*p* values
Age (years)	37.7 ± 12.8	35.2 ± 13.8	0.22 a
Gender (M/F)	14/20	19/13	0.14 b
Duration of illness (years)	6.7 ± 5.0	5.5 ± 5.2	0.16 a
Urine OB score	2.50 ± 0.66	2.50 ± 0.72	0.50 a
Proteinuria (g/g Cr)	1.48 ± 0.94	1.27 ± 0.86	0.18 a
Serum creatinine (mg/dL)	0.93 ± 0.38	0.94 ± 0.37	0.52 a
eGFR (mL/min/1.73 m^2^)	71.5 ± 24.6	77.8 ± 27.0	0.84 a
Serum IgA (mg/dL)	342.5 ± 121.4	319.6 ± 94.5	0.20 a
Histologic graded/II/III/IV)	11/13/7/3	11/14/6/1	0.35 b
No. of RAS inhibitor users/all	17/34	14/32	0.61 b

*p* value: a = *t* test, b = Chi-squared test

**Fig. 1:**
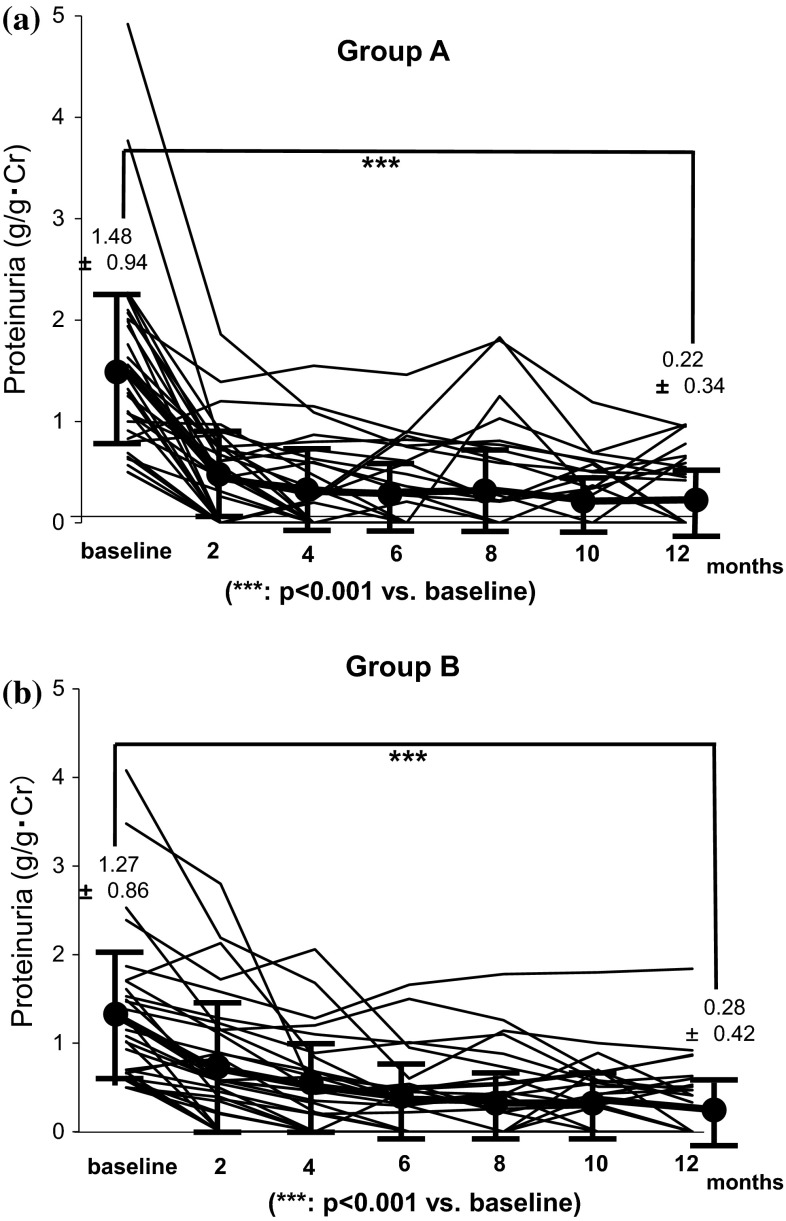
Variation in proteinuria at the start of the treatment and 2, 4, 6, 8, 10, and 12 months later. Mean level and standard deviation are also presented. In both groups, the proteinuria level decreased significantly at 12 months later compared with baseline

**Fig. 2:**
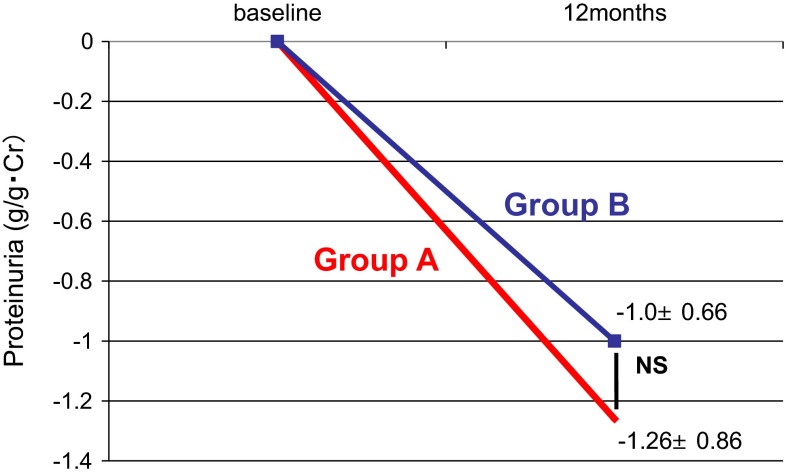
Comparison of the alleviation effect on proteinuria between Group A and Group B 12 months after treatment. No significant difference in the alleviation effect on proteinuria was found between groups

**Fig. 3:**
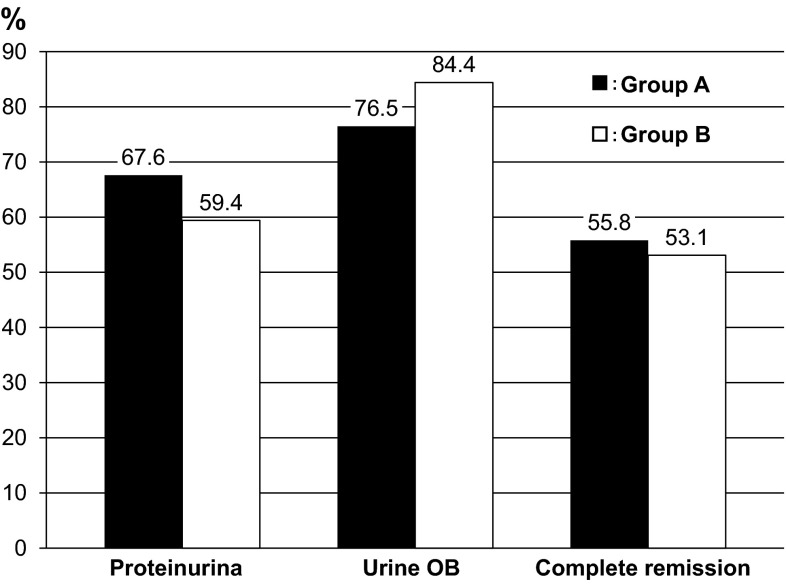
Remission rate of urinary findings 12 months after the initiation of the treatment. No significant difference in the remission rate of hematuria or proteinuria, or the complete remission rate of both parameters, was found between groups

**Fig. 4:**
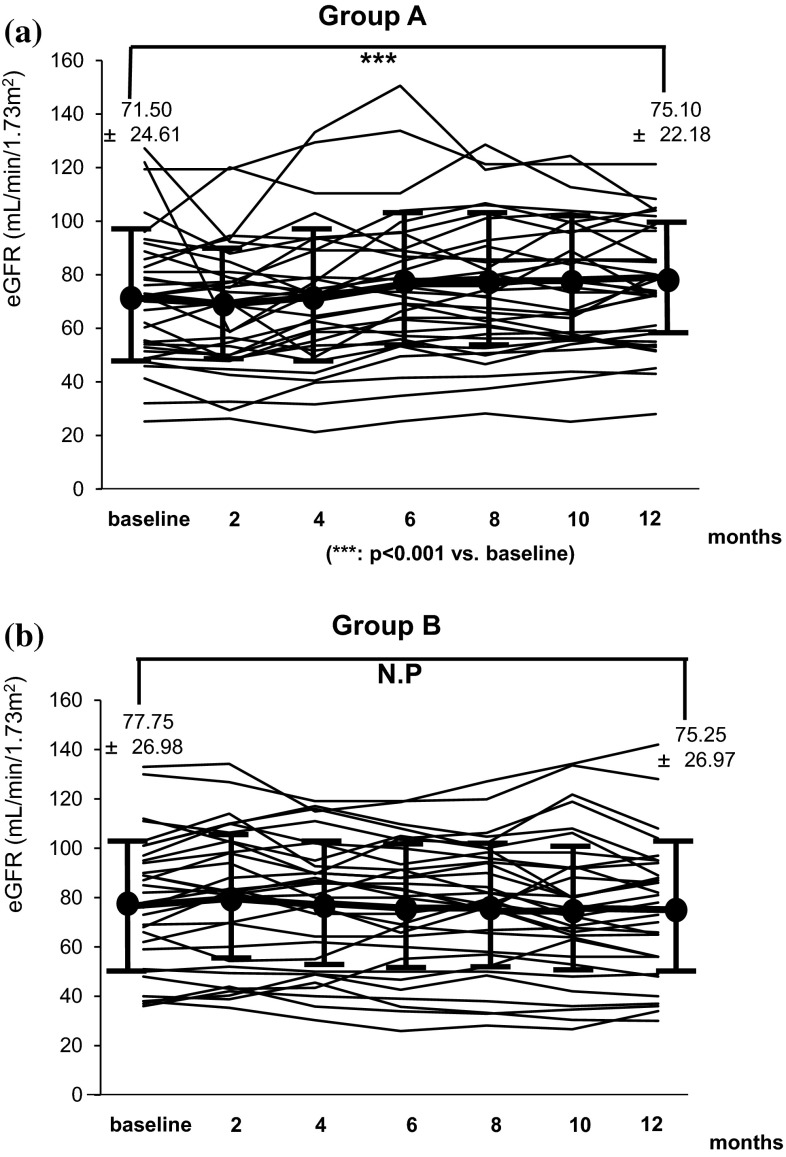
Variation in eGFR at the initiation of the treatment and 2, 4, 6, 8, 10, and 12 months later. Mean level and standard deviation are also presented. In Group A, the eGFR increased significantly 12 months after treatment compared with baseline (**a**). In Group B, no significant difference was found (**b**)

**Fig. 5:**
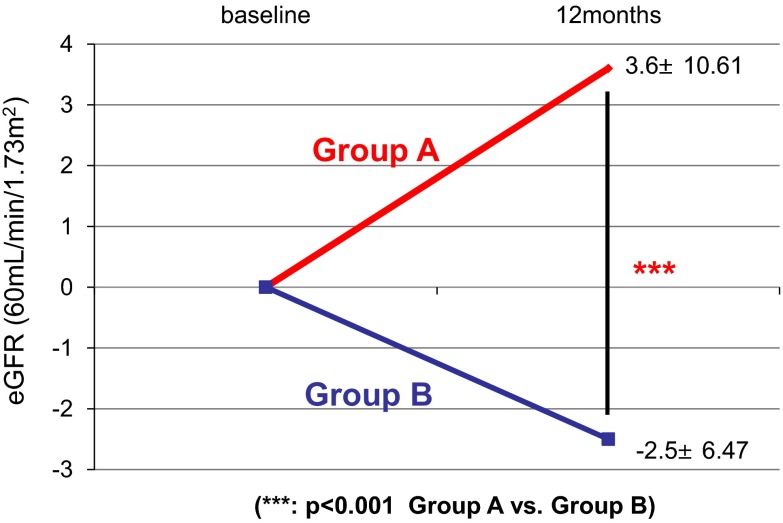
Comparison of changes in eGFR at the initiation of the treatment and 12 months later between Group A and Group B. Compared with Group B, a significant increase in eGFR was found in Group A

**Fig. 6:**
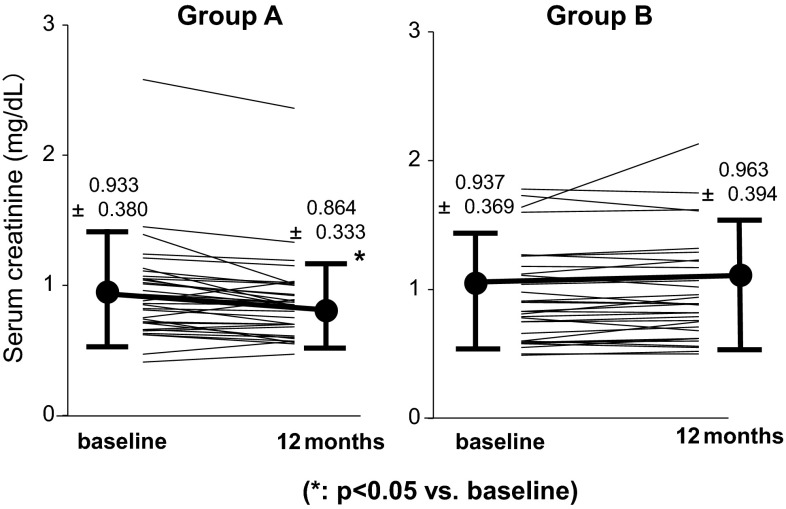
Variation in serum creatinine at the initiation of the treatment and 12 months. In Group A, serum creatinine decreased significantly 12 months after treatment compared with baseline. In Group B, no significant difference was found

Figure 1a, b shows variation in proteinuria at the start of the treatment and 2, 4, 6, 8, 10, and 12 months later. In both groups, significant reduction of proteinuria was found at 12 months later compared with baseline. No significant difference in the alleviation effect on proteinuria was found between groups (Fig. 2). For remission rates after 12 months, no significant difference in proteinuria and urinary occult blood was found between groups. The complete remission rates that both proteinuria and hematuria remitted were 55.8 % in Group A and 53.1 % in Group B, which were not significantly different (Fig. 3). For renal function, Group A showed a significant increase in eGFR at 12 months (71.5 ± 24.6 → 75.1 ± 22.2 mL/min/1.73 m^2^), whereas Group B tended to show its reduction (77.8 ± 27.0 → 75.3 ± 27.0 mL/min/1.73 m^2^) (Fig. 4a, b). For the improvement effects on eGFR, the effect in Group A was significantly superior (Fig. 5). In serum creatinine, Group A showed a significant decrease at 12 months (0.93 ± 0.38 → 0.86 ± 0.33 mg/dL), whereas Group B tended to show its increase (0.94 ± 0.37 → 0.96 ± 0.39 mg/dL) (Fig. 6).

**Fig. 7:**
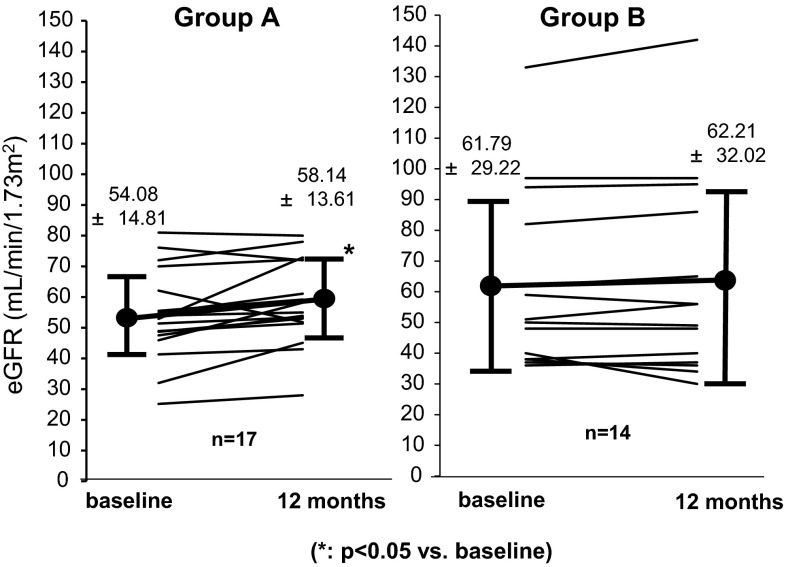
Variation in eGFR at the initiation of the treatment and 12 months after treatment about subgroups who were under RAS inhibitors. In 17 cases in Group A, eGFR increased significantly for 12 months before and after the treatment. Fourteen cases of Group B tended to increase for 12 months before and after the treatment, but there was no significant difference

We analyzed subgroups who were under RAS inhibitors (Fig. 7). In 17 cases in Group A, eGFR increased significantly for 12 months before and after the treatment (54.1 ± 14.8 → 58.1 ± 13.6 mL/min/1.73 m^2^). Fourteen cases of Group B tended to increase for 12 months before and after the treatment (61.8 ± 29.2 → 62.2 ± 32.0 mL/min/1.73 m^2^), but there was no significant difference.

**Fig. 8:**
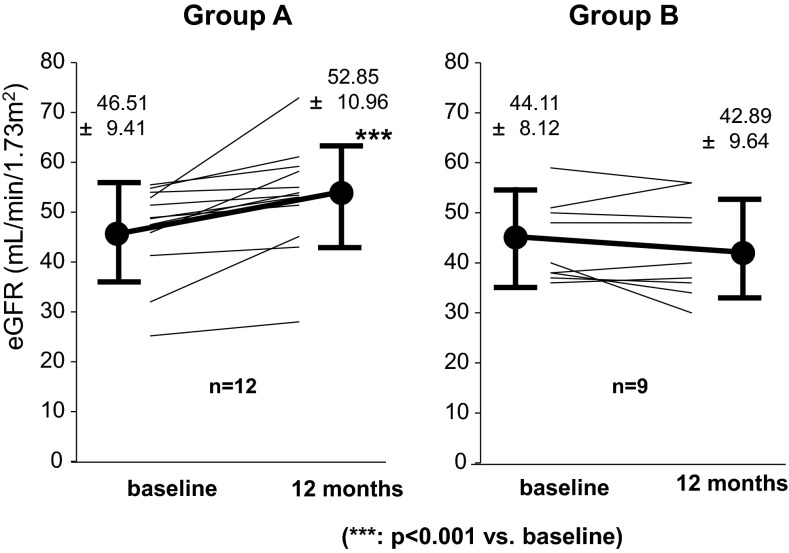
Variation in eGFR at the initiation of the treatment and 12 months after treatment about subgroups with eGFR < 60 mL/min/1.73 m^2^. All patients of both groups were taking RAS inhibitors. In 12 cases in Group A, eGFR increased significantly for 12 months before and after the treatment. Nine cases of Group B, conversely, tended to show reduction

In patients with eGFR < 60 mL/min/1.73 m^2^, evaluation was also conducted. In 12 cases in Group A, eGFR increased significantly for 12 months before and after the treatment (46.5 ± 9.4 → 52.9 ± 11.0 mL/min/1.73 m^2^). Nine cases of Group B, conversely, tended to show reduction (44.1 ± 8.1 → 42.9 ± 9.6 mL/min/1.73 m^2^) (Fig. 8). In all cases in Group A, eGFR increased after 12 months. In Group B, there were three cases where eGFR decreased by 5 % or higher after 12 months, the number of years going on after the initiation of the treatment exceeded 12 years in all the cases. In Group A, there were three cases where the number of years from disease onset until initiation of treatment exceeded 12 years and where eGFR increased by 5 % or higher at 12 months in all cases.

In both the groups, critical complications such as infections and avascular necrosis of the femoral head were absent during the course of the study. In both the groups, steroid acne requiring treatment was detected in several cases, but they were temporary and mild cases. In one case in Group B, administration of antidiabetes drugs was required during the course of treatment. No complications were associated with tonsillectomy, except for pain.

## Discussion

Immunosuppressive therapies after tonsillectomy for IgA nephropathy having active lesions were compared. In both groups, no significant difference was found in alleviation effects on proteinuria or remission rates of urinary findings 12 months after treatment. However, Group A showed a significant increase in eGFR. In cases which already had renal dysfunction at initiation of treatment, Group A showed a significant renal protective effect. In cases in Group B where treatment was ineffective, the number of years from the disease onset to initiation of the treatment tended to be high. The reason for similar remission rates and the improvement effect on the renal function obtained despite decreasing steroid dose was presumably because of the effect of combined MZR.

MZR specifically inhibits inosine monophosphate (IMP) dehydrogenase, a rate-controlling enzyme which is important for the synthesis of Guanosine monophosphate by lymphocytes such as mycophenolate mofetil (MMF), and selectively inhibits proliferation of lymphocytes, demonstrating an immunosuppressive effect. It is known that IMP dehydrogenase acts by chemically different mechanisms than MMF. MZR operates on both B lymphocytes and T cells so as to inhibit the immune reaction to T lymphocyte nondependent antigens and activate memory B cells and inhibit the synthesis of memory helper T cell and delay hypersensitive reaction. Moreover, recently it has been shown that MZR has further unique pharmacologic actions. It is reported that in the glomerular cells of IgA nephropathy, 14-3-3 protein and heat shock protein (HSP) 60 appear. It is shown that MZR combines with 14-3-3 protein or HSP60 to increase the transcription activity of glucocorticoid receptors and then enhance steroid effects [15, 16]. Based on these findings, we considered that the use of MZR in this study maintained its direct effect on the glomerular cells in IgA nephropathy when the steroid dose was decreased, and consequently, the remission rate in one-course pulse was equivalent to the three-course pulse.

For influence on the renal function, it is reported that mizoribine inhibited infiltration of macrophages into the glomerular and the interstitium and then inhibited glomerular sclerosis and interstitial fibrotic changes in a dose-dependent manner in insulin-nonresistant diabetic rats [17]. In a comparison study between steroid single therapy and steroid + MZR combined therapy in IgA nephropathy, steroid single therapy decreased proteinuria and increased sclerotic glomerulus, whereas the combined therapy decreased proteinuria and inhibited the increase in sclerotic glomerulus [18]. As its reason, it is presented because MZR inhibits the infiltration of macrophages to the renal interstitium and the expression of αSMA in muscle fibroblasts. Moreover, it is also reported that MZR inhibits the action of α3β1 integrin by reacting with HSP60, mentioned above, and then decreases the motion capability of fibroblast-specific protein (FSP)-1-positive fibroblasts which are involved in development of interstitial fibrous change in IgA nephropathy [19].

In addition, recently, it has been demonstrated that activated macrophages are deeply involved in the pathologic conditions of IgA nephropathy. Two lines of M1 and M2 act as macrophage activation modes. It is known that M1 is an inflammatory macrophage group activated by inflammatory cytokine such as IFN-γ and that M2 is another macrophage group activated by cytokines which have an anti-inflammatory effect and tissue repair functions such as interleukin (IL)-4 and IL-13. Ikezumi et al. [20] reported that in IgA nephropathy, M1-type activated macrophages are involved in formation of acute active lesions such as intratubular proliferation of the glomerulus or cellular crescent form and M2-type activated macrophages are involved in formation of chronic lesions such as glomerular sclerosis or interstitial fibrous change through syntheses of fibrous promotion factors such as TGF-β or CTGF. In a study using rat celiac macrophages, they also reported that steroids enhanced the expression of CD163, a marker of M2-type macrophages, and increased the syntheses of fibrous promotion factors and that the addition of MZR inhibited these [21]. As described above, MZR enhances the anti-inflammatory effect of steroids and also may inhibit the progress of chronic lesions such as fibrous tissue or sclerotic change by compensating for the negative effect of steroids.

As for progress of renal fibrous change, it is reported that activation of RAS as well as infiltration of macrophages plays an important role [22]. In IgA nephropathy, there have been some papers stating that RAS inhibitors have protective effects on the renal function [23–25]. In this study, there was no difference in the proportion of the patients using RAS inhibitors between both the groups, and additional administration was not conducted after the initiation of the treatment. Efficacy and safety of MZR combined with losartan in the treatment of IgA nephropathy were reported in RCT [26]. Recently, it has been reported that in rat models with unilateral ureteral obstruction, MZR combined with a direct renin inhibitor inhibits tubulointestinal fibrous change and inflammation [27]. These combined effects were significantly superior to individual effects. In this study, because RAS inhibitors were combined for all the patients with eGFR < 60 mL/min/1.73 m^2^, the combination of MZR and RAS inhibitors may enhance the renal protective effect.

Although harmful events observed during the follow-up observation period in this study were mild or slight, administration of antidiabetic drugs was required in one patient of Group B during the treatment course. It is reported that the incidence of diabetes induced by steroids ranges from 5 to 25 % and many of the cases occur within 1 year after the initiation of treatment [28]. In addition, steroids increase apoptosis of osteoblasts to inhibit osteogenesis and promote differentiation and activation of osteoclasts to promote bone absorption and then decrease the bone quantity [29]. These complications are reported to be dose dependent [30, 31].

It is known that because MZR has a selective inhibitory effect on proliferation of lymphocytes, the rate of side effects is lower than that of other immunosuppressive drugs [32]. Because MZR in this study was combined, the methylprednisolone pulse was conducted only once, and the total dose of steroids could be reduced considerably without diminishing the treatment effect. Further study should be conducted to investigate long-term results of this treatment.

We were aware of some limitations of this study. First, it was a single-center study with a low number of cases, and it was not a randomized controlled trial, which may have led to bias. Second, the follow-up period was too short to be able to assess several long-term outcomes, i.e., renal function. Third, Group B has slightly more males, and in Group B, there was less number of patients that took RAS inhibitors in comparison with Group A although these were not statistically significant. These might be responsible for the significant differences in the GFR changes partly. Therefore, we analyzed the subgroups who were under RAS blockade, and eGFR was increased for significant difference only in Group A. In patients with eGFR < 60 mL/min/1.73 m^2^, evaluation was also conducted. All patients of both groups were taking RAS inhibitors. In Group A, eGFR increased significantly, but in Group B, conversely, it tended to show reduction. However, the number of cases we analyzed decreased more, and evaluation about the other drugs was not done. Therefore, multicenter studies with large sample sizes and a long-term follow-up are needed to verify these results in the future.

## Conclusions

As an immunosuppressive therapy for IgA nephropathy, single-course steroid pulse + mizoribine combined therapy was superior to three-course steroid pulse therapy in protective effects on renal function. Single-course steroid pulse + mizoribine combined therapy is considered a viable treatment method for patients in whom the total dose of steroids must be reduced or in those where renal dysfunction and chronic lesions are present.
